# A Case Report on Pituitary Apoplexy Presenting as Acute Kidney Injury

**DOI:** 10.7759/cureus.3583

**Published:** 2018-11-13

**Authors:** Nidhi Shankar Kikkeri, Shivaraj Nagalli

**Affiliations:** 1 Neurology, University of Missouri, Columbia, USA; 2 Internal Medicine, Yuma Regional Medical Center, Yuma, USA

**Keywords:** hypotension, acute kidney injury, pituitary tumor, magnetic resonance imaging, cortisol

## Abstract

Pituitary apoplexy is characterized by the acute ischemic or hemorrhagic infarction of the pituitary gland. Acute kidney injury is a less recognized complication associated with pituitary apoplexy but occasionally can be a presenting condition in patients with pituitary apoplexy. Here we discuss the case of a patient who presented with significant volume depletion, admitted with acute kidney injury, and was later diagnosed with pituitary apoplexy.

## Introduction

Pituitary apoplexy (PA) is a medical emergency caused by the acute ischemic or hemorrhagic infarction of pituitary macroadenoma [1]. The term “pituitary apoplexy” was coined by Brougham in 1950, and the first index case was described by Bailey in 1898 [2]. Acute kidney injury (AKI) is a heterogeneous group of conditions characterized by a sudden decrease in the glomerular filtration rate, manifested by an increase in serum creatinine concentration or decrease in urine output. Acute kidney injury associated with pituitary apoplexy is rarely reported.

## Case presentation

A 73-year-old male with a medical history of hypertension and diabetes mellitus type 2, presented with complaints of vomiting for 4-5 days. Vomiting was non-bloody and non-bilious in nature and was associated with nausea and headache. The headache was sudden in onset, bifrontal, sharp in nature, non-radiating, and 8/10 in severity on a numerical rating pain scale (NRS). The patient also complained of constipation for 4-5 days. He denied associated abdominal pain or distension. There were no reports of associated visual disturbances, neck pain, neck rigidity, or fever. The patient also denied recent alcohol intake or unusual food intake prior to the onset of symptoms.

Initial vitals were remarkable for hypotension with blood pressure of 68/54 mm Hg which improved to 94/55 mm Hg after two liters of normal saline infusion. Physical exam was remarkable for dry mucosa but no focal neurological deficits were noted.

**Figure 1:**
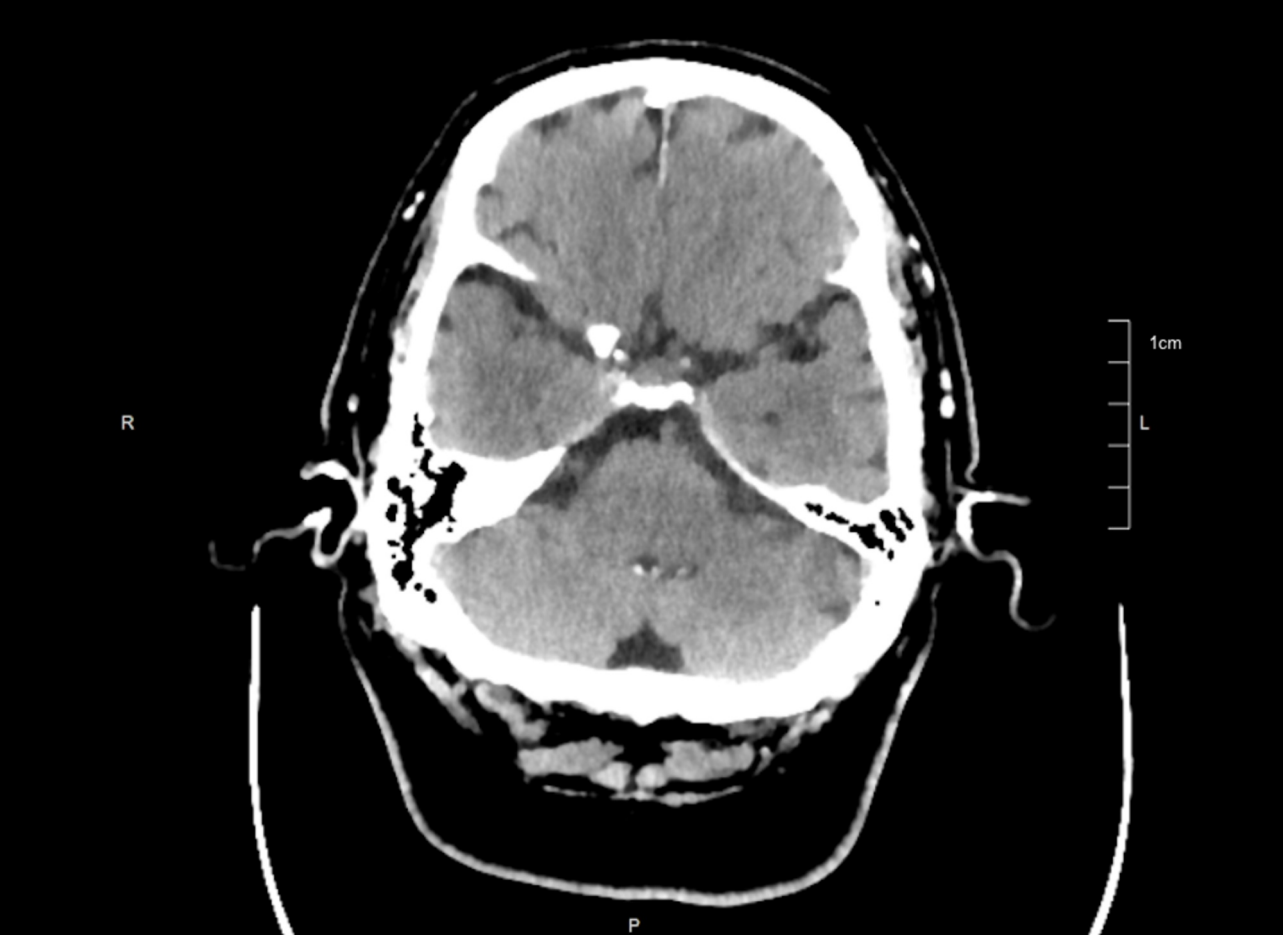
Computed tomography of the head showing no evidence of acute intracranial pathology

Computed tomography (CT) head was reported to be unremarkable with no evidence of acute intracranial pathologies as shown in Figure 1. Initial blood workup revealed hyponatremia with serum sodium of 128 mmol/L (normal: 137-145 mmol/L) and hypokalemia with serum potassium of 3.2 mmol/L (normal: 3.5-5.1 mmol/L). Serum bicarbonate was 27 mmol/L (normal: 22-30 mmol/L) and serum glucose was 171 mg/dL (normal: 74-105 mg/dL). Blood urea nitrogen and serum creatinine were 56 mg/dL (normal: 9-20 mg/dL) and 3.3 mg/dL (normal: 0.8-1.5 mg/dL), respectively.

**Figure 2:**
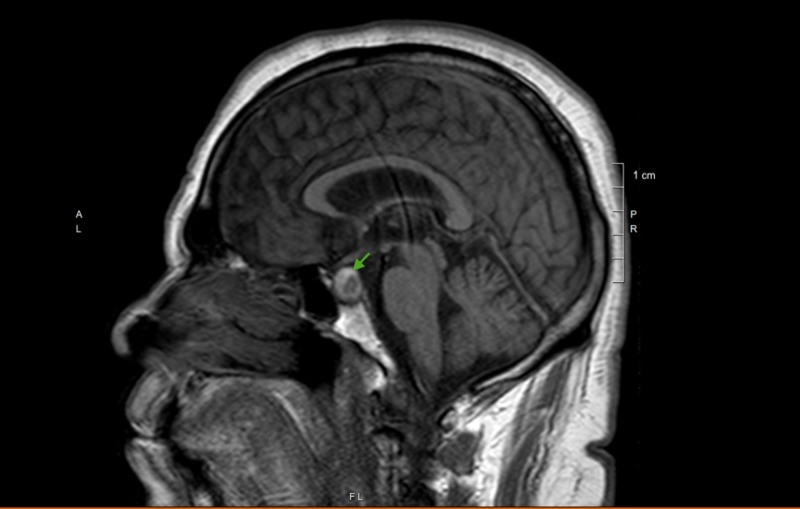
T1 sagittal section of magnetic resonance imaging of the brain showing pituitary mass with peripheral hyperintensity

The patient was admitted with an initial impression of acute kidney injury secondary to acute volume depletion resulting from vomiting. Aggressive hydration was initiated. Electrolytes were replaced. Over the next several hours, the hypotension resolved with the improvement in kidney function. However, the patient continued to report a persistent headache and nausea. Approximately 12-18 hours later, the patient started to report double vision with the development of left-sided ptosis. Magnetic resonance imaging (MRI) brain without contrast, as shown in Figure 2, revealed a pituitary mass of 1.2 cm x 1.3 cm x 1.2 cm size, arising from the left side of the pituitary gland within the sella, which was peripherally hyperintense on the T1 image. Also noted was the rightward displacement of the pituitary stalk with a mild extension of the tumor into the suprasellar cistern without displacement of the optic chiasm. Subsequent blood tests revealed an extremely low level of random serum cortisol at 0.69 mcg/dl (normal: 4.46-22.7 mcg/dl). Thyroid stimulating hormone (TSH) level was 0.3 mIU/ml (normal: 0.47-4.68 mIU/ml), free thyroxine (FT4) level was 1.4 ng/dL (normal: 0.78-2.19 ng/dL), and serum prolactin level was 2.6 ng/ml (normal: 3.7-17.9 ng/ml). Pituitary apoplexy was diagnosed and intravenous hydrocortisone was infused. Neurosurgery was consulted immediately and the patient was transferred to a tertiary care hospital for surgical decompression.

## Discussion

Pituitary apoplexy is a rare medical condition, with an estimated prevalence of nearly 6.2 cases per 100,000 inhabitants [3] and an incidence of 0.17 episodes per 100,000 person-years [4]. Pituitary apoplexy may be the first presentation of a previously undiagnosed pituitary macroadenoma in 70% of the cases [5].

Several theories have been suggested for the mechanisms of pituitary apoplexy. One of them suggests that PA is due to ischemia and infarction followed by hemorrhage when the pituitary tumor outgrows its blood supply [6]. Another one points to the compression of trabecular branches of superior hypophyseal arteries due to the outgrowth of the pituitary tumor resulting in ischemic necrosis [6]. Several triggering factors like coagulation abnormalities and changes in vascular flow to the pituitary gland have been suggested in the pathogenesis of PA [7].

According to Kidney Disease Improving Global Outcomes (KDIGO), AKI is defined as any of the following: increase in serum creatinine by more than or equal to 0.3 mg/dl (≥ 26.5 umol/l) within 48 hours; or an increase in serum creatinine to more than or equal to 1.5 times the baseline, which is known or presumed to have occurred within the preceding seven days; or a urine volume less than 0.5 ml/kg/h for six hours [8]. The etiologies of AKI can be broadly categorized into pre-renal (decreased renal perfusion), intrinsic renal (ischemic and toxic damage to the kidneys), and post-renal (obstruction to the urinary outflow).

Headache is the most common symptom reported in patients with PA followed by nausea and vomiting [1,9]. The origin of the headache is unclear but is thought to be due to meningeal irritation, or the stretching of dura mater or the trigeminal nerve within the cavernous sinus [9-10]. Other symptoms include visual field defects, decreased visual acuity, photophobia and ophthalmoplegia, a decrease in the level of consciousness, symptoms of pituitary hormonal deficiencies, and cranial nerve palsies [1,11].

Anterior pituitary hormonal deficiencies are seen in about 80% of patients with PA. Hypotension seen in patients with PA is explained by the hypocortisolism resulting from the deficiency of adrenocorticotrophic hormone (ACTH) which is seen in 70% of patients with PA. Other endocrine dysfunctions include growth hormone (GH) deficiency (88%), hypothyroidism (50%), and hypogonadotropic hypogonadism (75 %) [11-13]. Elevated prolactin level can be seen due to prolactinoma or due to the lack of a inhibitory hormone from the hypothalamus [14]. Low prolactin levels can also be seen, as in our case, due to the increase in intrasellar pressure and are less likely to recover from hypopituitarism following surgical decompression [10].

In our case, acute kidney injury resolved after rehydration and supportive care. However, headache and nausea persisted. Further, with the development of double vision, MRI brain was considered which was suggestive of pituitary apoplexy. Laboratory tests were confirmatory and helped in establishing the diagnosis.

As in our case, CT head is known to be less sensitive than MRI in diagnosing PA. MRI brain is superior to CT head in identifying pituitary tumors (100% vs 93%) and hemorrhage (88% vs 21%) and remains the gold standard in diagnosing PA [13,15-17].

Hypotension is a well-documented risk factor for acute kidney injury. However, acute kidney injury is a less recognized complication in patients with PA. The likely mechanism of acute kidney injury in this patient is hypotension due to the deficiency of corticosteroids resulting from PA and acute volume depletion due to excessive vomiting. Renal vasoconstriction in patients with systemic hypotension and true volume depletion results in decreased renal perfusion and thereby decrease in the glomerular filtration rate.

## Conclusions

Pituitary apoplexy presenting as acute kidney injury is rarely reported. A detailed history and evaluation by the clinicians with an emphasis on the etiologies of the presenting complaints are necessary to identify such medical emergencies.
